# *Pinus thunbergii* Parl. Extracts Reduce Acute Inflammation by Targeting Oxidative Stress

**DOI:** 10.1155/2021/7924645

**Published:** 2021-01-13

**Authors:** Chan Jong Yoon, Won Seok Choi, Hyun Sik Kang, Hong Jo Kim, Wang Tae Lee, Jong Seok Lee, Sarah Lee, Su Young Son, Choong Hwan Lee, Uy Dong Sohn, Ji Yun Lee

**Affiliations:** ^1^College of Pharmacy, Chung-Ang University, Seoul 06974, Republic of Korea; ^2^National Institute of Biological Resources, Incheon 22689, Republic of Korea; ^3^Department of Bioscience and Biotechnology, Konkuk University, Seoul 05029, Republic of Korea; ^4^Research Institute for Bioactive-Metabolome Network, Konkuk University, Seoul 05029, Republic of Korea

## Abstract

*Pinus thunbergii* Parl. (PTP) has traditionally been used for edible and medicinal purposes to treat several disorders, including diabetes and neuralgia. Therefore, this study sought to evaluate the inhibitory effects of PTP leaf ethanol extracts on acute inflammation. Moreover, the reactive oxygen species (ROS) scavenging activity, superoxide dismutase (SOD) activity, lipopolysaccharide (LPS)-induced nitric oxide (NO) generation, and H_2_O_2_-induced lipid peroxidation capacity of PTP were assessed *in vitro* in RAW 264.7 macrophages. Our results suggest that PTP prevents cell damage caused by oxidative free radicals and downregulates the expression of LPS-induced inflammation-associated factors including inducible nitric oxidase synthetase (iNOS), cyclooxygenase-2 (COX-2), and prostaglandin E_2_ (PGE_2_). PTP inhibited NO production by 53.5% (*P* < 0.05) and iNOS expression by 71.5% (*P* < 0.01) at 100 *µ*g/mL. PTP at 100 *µ*g/mL also inhibited ROS generation by 58.2% (*P* < 0.01) and SOD activity by 29.3%, as well as COX-2 expression by 83.3% (*P* < 0.01) and PGE2 expression by 98.6% (*P* < 0.01). The anti-inflammatory effects of PTP were confirmed *in vivo* using an arachidonic acid (AA)-induced ear edema mouse model. Ear thickness and myeloperoxidase (MPO) activity were evaluated as indicators of inflammation. PTP inhibited edema formation by 64.5% (*P* < 0.05) at 1.0 mg/ear. A total of 16 metabolites were identified in PTP extracts and categorized into subgroups, including two phenolic acids (mainly quinic acid), seven flavonoids, five lignans, one sesquiterpenoid, and one long-chain fatty acid. Therefore, our results suggest that PTP possesses anti-inflammatory properties.

## 1. Introduction

The superoxide anion (·O^2-^), the first reactive oxygen species (ROS) produced during normal metabolism, is converted to a secondary ROS (e.g., hydrogen peroxide, hydroxyl radical, or peroxynitrite) that can cause damage to intracellular nucleic acids and proteins. Previous studies demonstrated that ROS accumulation can lead to increased levels of proinflammatory factors [1]. ROS have been recently recognized as intermediate signaling mediators, and the ROS inducer lysophosphatidic acid can promote proliferative signaling through the generation of second messengers [2].

Inflammation is among the most important defense responses against invading pathogens [3]. Specifically, lipopolysaccharide (LPS) is an inflammatory stimulus that induces a strong immune response by activating macrophages, which are essential for the release of inflammatory mediators in response to various adverse stimuli [4]. The levels of nitric oxide (NO) and prostaglandin E_2_ (PGE_2_) released by macrophages and monocytes are typically increased during the inflammatory reaction. Because NO is a free oxygen radical, the pathological process of inflammation can result in cytotoxicity, especially in inflammatory diseases [5]. The generation of free oxygen radicals (e.g., NO) is caused by an increase in the levels of inducible nitric oxide synthase (iNOS) and cyclooxygenase-2 (COX-2) [6]. Furthermore, inflammatory factors such as cytokines and bacterial LPS can result in increased iNOS in macrophages, leading to the generation of intracellular ROS [7, 8]. Some studies have demonstrated that the overexpression of iNOS or COX-2 may contribute to disease-related inflammation [8]. In inflamed tissue, LPS-activated macrophages play a major role in the production of proinflammatory cytokines, including tumor necrosis factor (TNF)-*α*, NO, and PGE_2_ [9]. Among these, TNF-*α* produced by macrophages is a proinflammatory mediator involved in acute and chronic inflammatory reactions [10].

Symptoms of inflammation include redness, swelling, and itching. In the present study, we focused on edema as an indicator of *in vivo* inflammation in the ear. Ear edema models in mice are sensitive and reliable indicators for the anti-inflammatory activity of topically applied materials. This model is also useful for other types of analyses, including the measurement of tissue cytokine levels, myeloperoxidase (MPO) analysis, and histopathological analysis, once the basic anti-inflammatory responses are displayed [11].

*Pinus thunbergii* Parl. (PTP) is more commonly known as Japanese black pine due to the black coloration of its leaves and stems. This plant is known as “gomsol” in Korea, “hēisōng” in China, and “kuromatsu” in Japan [12]. Both the pollen and bark of PTP are edible and have traditionally been used in oriental medicine for various therapeutic purposes. PTP extracts are known to exhibit antioxidant, antibacterial, antidiabetic, anticardiovascular, and anti-inflammatory properties; however, its major active compounds have not been characterized [13].

Therefore, this study sought to investigate the detailed mechanisms by which PTP pine needle ethanol extract exerts its anti-inflammatory effects *in vitro* and *in vivo.* To achieve this, we evaluated free radical generation during inflammatory responses and analyzed the effects of PTP on the inflammatory response modulated by the expression of COX-2, iNOS, and other proinflammatory cytokines.

## 2. Materials and Methods

### 2.1. Preparation of PTP Needle Extracts

PTP pine needles were collected in Gangjin-gun, Jeollanam-do, Republic of Korea, in August 2014. The PTP needles (104.0 g) were extracted 3 times with 70% ethanol at room temperature for 24 h and then filtered. The extracts were then concentrated under reduced pressure at 45°C using a rotary evaporator, after which the concentrated extracts were lyophilized. The yield was 10.86 g (10.44%). The extract stock solutions were prepared in dimethyl sulfoxide (DMSO, Sigma, St. Louis, USA) at a 20 mg/mL concentration. A voucher specimen (NIBRVP0000519852) of the authenticated plant material was deposited in the Wildlife Natural Products Bank of the Biological and Genetic Resources Utilization Division.

### 2.2. Cell Culture

The RAW 264.7 cell line is a mouse monocyte-macrophage cell line that was established from the ascites of a tumor induced in a male mouse. RAW 264.7 cells were purchased from ATCC® and were cultured in Dulbecco's modified Eagle's medium (DMEM) (Welgene, Republic of Korea) supplemented with 10% fetal bovine serum (FBS) (Welgene, Republic of Korea) and 1% antibiotic antimycotic at 37°C and 5% CO_2_ [6]. All experiments were conducted in accordance with the Chung-Ang University Code of Ethics.

### 2.3. Measurement of Cytotoxicity of PTP

Cytotoxicity was determined using the 3-(4,5-dimethylthiazol-2-yl)-2,5-diphenyltetrazolium bromide (MTT) conversion assay. The MTT method involves the conversion of MTT to colored formazan via intact mitochondria. The MTT assay relies on absorbance to quantify the cells present in a given sample [14]. To investigate the cytotoxicity of PTP, RAW 264.7 cells (1 × 10^4^ cells/well) were plated in duplicate in 96-well plates and treated with three PTP extract concentrations (25, 50, and 100 *μ*g/mL) for 24 h. MTT was added to the culture medium at a final concentration of 0.5 mg/mL. Absorbance was measured at 570 nm with a microplate reader (FlexStation 3, Molecular Devices, Sunnyvale, CA, USA).

### 2.4. Measurement of LPS-Induced NO Production

RAW 264.7 cells were cultured in a 96-well plate and incubated for 18 h at 37°C in 5% CO_2_. The cells were then incubated for an additional 18 h after the addition of 0.1 *µ*g/mL LPS (Sigma, St. Louis, MO, USA). NO production was determined with the Griess reagent (100 *µ*L; 0.1% naphthylethylenediamine and 1% sulfanilamide in 5% H_3_PO_4_ solution) (Sigma, St. Louis, MO, USA). The Griess reagent was added to 100 *μ*L of each of the supernatants obtained from the cell samples. After 10 min of a light-protected incubation period, the amount of NO was measured at 550 nm with a microplate reader (FlexStation 3, Molecular Devices, Sunnyvale, CA, USA). The NO contents were then quantified with a sodium nitrite serial dilution standard curve as previously described [15].

### 2.5. LPS-Induced iNOS, COX-2, and SOD Expression

RAW 264.7 cells were cultured in DMEM with 10% FBS in 6-well plates and incubated with three PTP concentrations (25, 50, and 100 *µ*g/mL). The cells were then stimulated with 0.1 *µ*g/mL LPS and incubated for an additional 18 h. After incubation, the cells were washed twice with PBS and lysed in lysis buffer (RIPA buffer, 100 mM vanadate, 100 mM PMSF). After 30 min, cell lysates were obtained by centrifugation at 10,000 rpm for 20 min at 4°C. Total protein was measured with the Thermo BCA protein assay using bovine serum albumin as a standard. Samples containing equal protein concentrations were separated by 10% sodium dodecyl sulfate-polyacrylamide gel electrophoresis at 100 V and transferred to PVDF membranes. Nonspecific binding was blocked with TBS-T (1 M Tris-HCl, pH 7.6, 2.5 M NaCl, and 0.5% Tween 20) containing 5% bovine serum albumin for 1 h at room temperature [16]. The membranes were incubated overnight at 4°C with primary antibodies (iNOS, COX-2, SOD1, and SOD2, Cell Signaling, MA, USA, or *β*-actin, Santa Cruz, Texas, USA) diluted in TBS-T (1 : 1000), then washed with TBS-T, and incubated with secondary antibodies for 2 h at room temperature. The protein bands and quantitative data were analyzed [17] using the Bio-Rad ChemiDoc system and Quantity One software (Bio-Rad Chemical Division, Richmond, CA, USA).

### 2.6. LPS-Induced Proinflammatory Cytokines and PGE_2_ Expression

TNF-*α*, IL-6, IL-1*β*, and PGE2 expression in the cultured cell supernatants was quantified using an enzyme-linked immunosorbent assay (ELISA) kit (Quantikine® ELISA, R&D Systems, MN, USA) [18] according to the manufacturer's instructions. Absorbance was measured at 450 nm using a FlexStation 3 microplate reader (Molecular Devices, USA).

### 2.7. ROS Scavenging Activity

An H_2_DCF-DA assay was performed to detect intracellular ROS formation. RAW 264.7 macrophages were cultured for 24 h and pretreated with PTP (25, 50, and 100 *μ*g/mL) for 3 h. Afterward, 0.1 *μ*g/mL LPS was added to each well and incubated for 24 h. The macrophages were then treated with 20 *μ*M of H_2_DCF-DA for 30 min. ROS were detected with a fluorescence microplate reader (FlexStation 3, Molecular Devices, USA) at 485 and 535 nm excitation and emission wavelengths, respectively. Representative fluorescence images were obtained using a Leica DM 480 camera (Leica, Wetzlar, Germany).

### 2.8. LPS-Induced Superoxide Dismutase Activity

SOD expression in the cultured cell lysates was quantified using an ELISA kit. Cells were cultured for 24 h in a 24-well plate and pretreated with PTP (25, 50, and 100 *μ*g/mL) for 3 h, after which they were treated with 0.1 *μ*g/mL LPS for 24 h. After washing with PBS, 400 *μ*L of RIPA was added to each well for 5 min. The lysates were centrifuged at 12,000 g for 15 min, and the supernatants were collected to measure the SOD concentration. ELISA kits for SOD (Cloud‐Clone Corp., Katy, TX, USA) were used according to the manufacturer's protocol.

### 2.9. Lipid Peroxidation Assay

Lipid peroxidation was used to measure malondialdehyde (MDA) production to determine peroxidation. MDA was then diluted to a final 100 *µ*L volume. Briefly, 200 *µ*L of thiobarbituric acid (TBA) was added to each sample, followed by incubation at 95°C for 60 min [19]. The samples were then cooled in an ice bath for 10 min, after which 300 *μ*L of n-butanol were added to the stock solution and centrifuged at 16,000 g for 3 min. MDA-TBA was added to the samples in a 96-well plate, and absorbance was measured at 532 nm using a microplate reader. MDA was then quantified using an MDA standard.

### 2.10. Animals

ICR mice (female, 7 weeks old) were obtained from Young Bio (Young Bio Inc., Korea) and kept in the animal facility of Chung-Ang University at room temperature with free access to water and food. All experiments were approved by and performed in accordance with the Institutional Animal Care Use Committee of Chung-Ang University (#IACUC-2020-00135).

### 2.11. Acute Inflammation Model

To assess anti-inflammatory activity during acute inflammation, ear swelling assays were conducted in mouse models as described by Inoue et al. [20]. This approach is considered a relatively reliable method for *in vivo* anti-inflammatory activity assessment [19, 21]. The samples were administered with a pipette (20 *μ*L/ear) to the inside and outside of the right ear of each mouse. DMSO was used as a vehicle and was therefore used as a negative control. After 10 min, arachidonic acid (AA) (1 mg/ear) was applied to the right ear at the same site. After edema formation by AA administration, ear thickness was measured using a micrometer (Mitutoyo Mfg., Japan). For histologic analyses, lung tissue samples were fixed with 10% neutral phosphate-buffered formalin and embedded in paraffin using a Tissue-Tek® TEC™ 5 Tissue Embedding Console System (Sakura Finetek®, Torrance, CA, USA). The embedded lung tissue samples were sectioned at a 4 *μ*m thickness and then stained with hematoxylin and eosin (H & E) to assess the severity of the ear edemas.

### 2.12. Assessment of MPO Activity in AA-Induced Inflamed Ear Tissue

To quantify the infiltration of inflammatory cells (neutrophils) in inflammatory tissues, myeloperoxidase (MPO), an enzyme that catalyzes intracellular oxidation reactions, was measured using hydrogen peroxide as a substrate (i.e., TMB colorimetric assay designed by Suwendi et al. [22]). Briefly, 30 *μ*L of prepared tissue homogenate was added to a 96-well microplate, after which 200 *μ*L of reaction buffer was incorporated. The samples were incubated at 37°C for 3 min. Then, 30 *μ*L of 1.46 mM sodium acetate buffer (pH 3.0) was added to terminate the reaction. Absorbance was measured at 620 nm using a microplate reader. A standard curve was then created using an MPO standard to calculate the MPO activity of each sample by comparing its absorbance to that of the sample reaction solution alone.

### 2.13. UHPLC-Q-Orbitrap-MS of PTP Extracts

PTP extract samples were analyzed by UHPLC-Q-Orbitrap-MS using a Q-Exactive Orbitrap mass spectrometer equipped with an electrospray interface (Thermo Fisher Scientific, San José, CA) coupled with a Dionex UltiMate 3000 RS Column Compartment, RS pump, and RS autosampler (Dionex Corporation, Sunnyvale, CA). The extracted samples were separated with a Hypersil GOLD C18 selectivity LC column (i.d., 1.9 *μ*m, 50 × 2.1 mm, Thermo Fisher Scientific) at a column oven temperature of 25°C. The mobile solvent, gradient flow program, and other parameters were implemented as described in a previous study [23]. Dried PTP extract was redissolved in 70% ethanol and then analyzed.

### 2.14. Statistical Analyses

All data were reported as the mean ± standard deviation (SD) of at least five independent experiments. Significant differences between groups were identified via Student's t-test and one-way ANOVA. *P* values <0.05 were considered statistically significant.

## 3. Results and Discussion

### 3.1. Cytotoxicity of PTP in RAW 264.7 Macrophages

**Figure 1:**
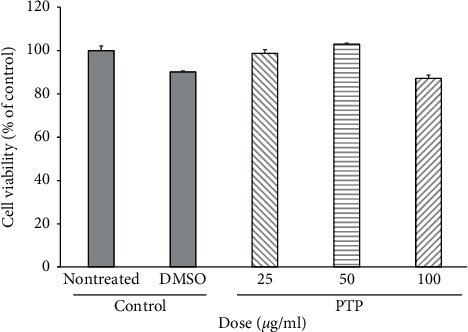
Cytotoxicity of PTP on RAW 264.7 macrophage cells. Cell viability was measured using the MTT colorimetric assay. All values are presented as the mean ± SD; PTP: *Pinus thunbergii* Parl.

The MTT assay was performed to determine the cytotoxicity of PTP towards cells. Cells were treated with three concentrations of PTP (25, 50, and 100 *μ*g/mL) for 24 h. No significant differences in cell viability were observed regardless of PTP concentrations compared with that of the control group, suggesting that the doses tested herein were not toxic to macrophages (Figure 1). Moreover, we confirmed that PTP only causes cytotoxicity when administered for over 24 h. Therefore, our results demonstrate that neither of the PTP concentrations tested herein rendered toxic effects, thus highlighting the potential of PTP as an anti-inflammatory agent that does not cause direct cell damage.

### 3.2. Effect of PTP on LPS-Induced NO and iNOS Production in RAW 264.7 Macrophages

**Figure 2:**
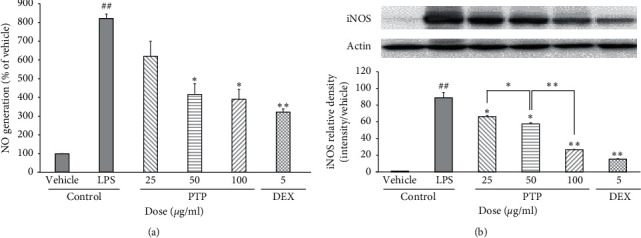
Effect of PTP on LPS-induced NO (a) and iNOS (b) production in RAW 264.7 macrophages. The NO generation values are reported as % activity relative to the control. To measure iNOS production, cells were pretreated with PTP (25, 50, and 100 *μ*g/ml) or DEX (5 *μ*g/mL), then treated with LPS (0.1 *μ*g/mL). iNOS relative density was analyzed using densitometry. All values are reported as the mean ± SD. # indicates a significant difference from the negative control group (##*P* < 0.05). ^*∗*^ indicates significant difference from the positive control group (^*∗*^*P* < 0.05, ^*∗∗*^*P* < 0.01). PTP: *Pinus thunbergii* Parl.; DEX: dexamethasone.

NO production is known to increase with the iNOS inflammatory reaction [24]. LPS increased NO production approximately 8-fold in the positive control group, whereas PTP reduced NO production in a concentration-dependent manner. PTP inhibited NO production by 24.6%, 50.0%, and 53.5% at 25, 50, and 100 *µ*g/mL, which constituted a substantial reduction in LPS-induced NO production. At the highest PTP concentration, the amount of NO production was reduced to the level achieved with dexamethasone (DEX) (Figure 2(a)). Protein levels of iNOS were 88 times higher in the LPS treatment group than that in the vehicle control group (*P* < 0.01). Similar to the NO production results, pretreatment with PTP significantly reduced LPS-induced iNOS expression in a dose-dependent manner. Specifically, PTP inhibited iNOS expression by 25.0%, 36.3%, and 71.5% at 25, 50, and 100 *μ*g/mL, respectively (Figure 2(b)).

### 3.3. Effect of PTP on Oxidative Stress and Malondialdehyde Production in RAW 264.7 Macrophage Cell Membranes

**Figure 3:**
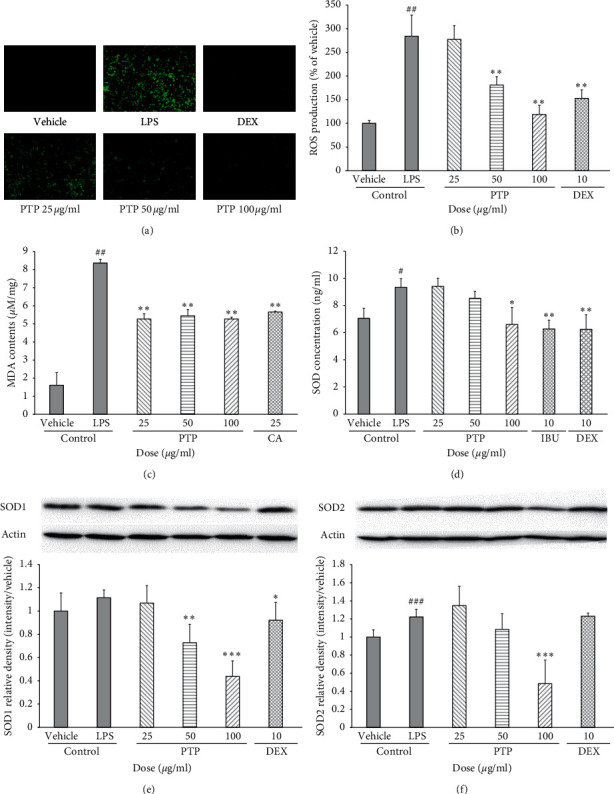
Effect of PTP on oxidative stress in RAW 264.7 macrophages. To measure ROS and SOD concentration, the cells were pretreated with PTP (25, 50, and 100 *μ*g/mL), DEX (10 *μ*g/mL), or IBU (10 *μ*g/mL), then treated with LPS (0.1 *μ*g/mL). ROS production was detected with a fluorescence microplate reader (Ex. 485 nm; Em. 535 nm) (a), (b). To measure MDA contents, 200 *μ*L of thiobarbituric acid (TBA) was added to each sample, after which absorbance was measured at 532 nm (c). SOD concentration was measured with an ELISA kit (d). SOD1 and SOD2 production were measured by western blot (e), (f). SOD1 and SOD2 relative density was analyzed using densitometry. All values were reported as the mean ± SD. # indicates a significant difference from the negative control group (#*P* < 0.05, ##*P* < 0.01, ###*P* < 0.001). ^*∗*^ indicates a significant difference from the positive control group (^*∗*^*P* < 0.05, ^*∗∗*^*P* < 0.01, ^*∗∗∗*^*P* < 0.001). PTP: *Pinus thunbergii* Parl.; DEX: dexamethasone; IBU: ibuprofen; CA: caffeic acid.

The effect of PTP on ROS generation was also measured (Figures 3(a) and 3(b)). Similar to our previous findings, PTP inhibited ROS generation in a dose-dependent manner by 36.2% and 58.2% at 50 and 100 *µ*g/mL, respectively. MDA contents representing lipid peroxidation were increased by LPS. The effect of PTP was similar to that of caffeic acid as a positive control at the lowest PTP concentration (25 *μ*g/mL) (Figure 3(c)). The effect of PTP on SOD concentration was characterized via ELISA (Figure 3(d)). PTP significantly inhibited SOD activity at 100 *µ*g/mL. We also measured SOD1 and SOD2 levels by western blot (Figures 3(e) and 3(f)). Although SOD1 levels were not significantly increased in the LPS group, 50 and 100 *µ*g/mL of PTP significantly decreased the SOD1 level. On the other hand, SOD2 levels were significantly increased in the LPS group. This elevation of SOD2 levels was significantly decreased by 100 *µ*g/mL of PTP. These differences in the reactivity of SOD1 and SOD2 to LPS were consistent with a previous study, suggesting that there was a selective SOD2 induction in response to LPS with no effect on SOD1 [25]. These results suggest that the anti-inflammatory effects of PTP extracts may be mediated by ROS scavenging. Hydrogen peroxide (H_2_O_2_), one of the main ROS produced during intracellular inflammation, is known to be an important causative factor in the inflammatory response due to its ability to move across the cell membrane [26]. This ROS can be deleterious to cells, as it can inactivate enzymes and receptors, disrupt cellular connective tissue, and lead to cell injury. These results suggest that PTP possesses intracellular ROS-superoxide radical-scavenging activity, which was demonstrated by the inhibition of superoxide anion generation by PTP.

### 3.4. Effect of PTP on COX-2 and PGE_2_ Expression

**Figure 4:**
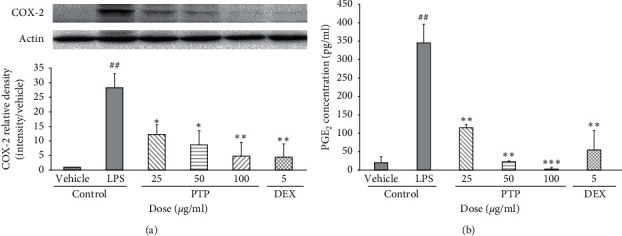
Effect of PTP on COX-2 (a) and PGE_2_ (b) expression. To measure COX-2 production, cells were pretreated with PTP (25, 50, and 100 *μ*g/mL) or DEX (5 *μ*g/mL), then treated with LPS (0.1 *μ*g/mL). COX-2 relative density was analyzed using densitometry. The PGE_2_ concentration values are reported as % activity relative to the negative control group. All values are reported as the mean ± SD. # indicates a significant difference from the negative control group (##*P* < 0.01). ^*∗*^ indicates a significant difference from the positive control group (^*∗*^*P* < 0.05, ^*∗∗*^*P* < 0.01, ^*∗∗∗*^*P* < 0.001). PTP: *Pinus thunbergii* Parl.; DEX: dexamethasone.

COX-2 protein levels in response to LPS treatment were 28 times higher than those in the control group (*P* < 0.01). Similar to our observations of NO and iNOS production, we observed that PTP inhibited LPS-induced COX-2 expression in a dose-dependent manner by 58.2%, 72.5%, and 83.3% at 25, 50, and 100 *μ*g/mL, respectively. The effect was similar to that of the dexamethasone group at the highest PTP concentration (100 *μ*g/mL) (Figure 4(a)). Pretreatment with PTP significantly reduced the macrophage expression of PGE_2_ stimulated by LPS in a dose-dependent fashion. Specifically, PTP inhibited PGE_2_ expression by 66.6%, 92.8%, and 98.6% at 25, 50, and 100 *μ*g/ml, respectively (Figure 4(b)).

Furthermore, PTP inhibited the expression of proinflammatory mediators such as iNOS and COX-2, which were induced by LPS. Histological studies have shown that enzymes such as COX-2 synthesize PGE_2_ in endothelial cells upon exposure to inflammatory stimuli [27]. iNOS and COX-2 are two important inducible enzymes responsible for the production of NO and PGE_2_, and both of which are important inflammatory mediators [28]. Inhibiting the expression of iNOS and COX-2 to reduce the production of NO and PGE_2_, which are components of the native immune system, can be thus used as a therapeutic strategy to treat inflammatory diseases [29].

### 3.5. Effect of PTP on TNF-*α* Expression

**Figure 5:**
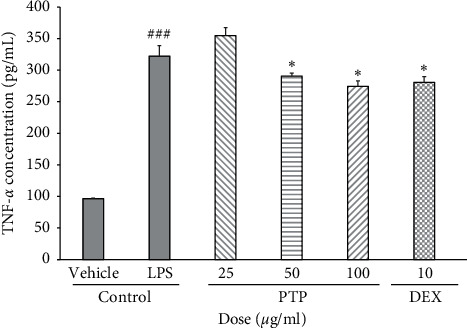
Effect of PTP on TNF-*α* expression. To measure TNF-*α* production, cells were pretreated with PTP (25, 50, and 100 *μ*g/mL) or DEX (10 *μ*g/mL), then treated with LPS (0.1 *μ*g/mL). All values represent the mean ± SD. # indicates a significant difference from the negative control group (###*P* < 0.001). ^*∗*^ indicates a significant difference from the positive control group (^*∗*^*P* < 0.05). PTP: *Pinus thunbergii* Parl.; DEX: dexamethasone.

TNF-*α* is a cytokine that activates inflammatory and apoptotic signals [30]. Therefore, we estimated the effects of PTP on the expression of TNF-*α*. Pretreatment with 50 and 100 *μ*g/mL of PTP significantly reduced TNF-*α* expression by 9.8% and 14.8%, respectively (Figure 5). The expression levels of other proinflammatory cytokines (IL-1*β* and IL-6) were also determined and there were no significant differences between groups (data not shown).

TNF-*α* is a cytokine that induces ROS through endothelial mitochondria and NAD(P)H at the plasma membrane. Furthermore, TNF-*α* can increase nitric acid levels, which under certain circumstances produces perinitrate [31]. PTP inhibited LPS-induced TNF-*α* expression, which was likely associated with a decrease in ROS and SOD expressions.

### 3.6. Effect of PTP on an Acute Inflammatory Edema Model *In Vivo*

**Figure 6:**
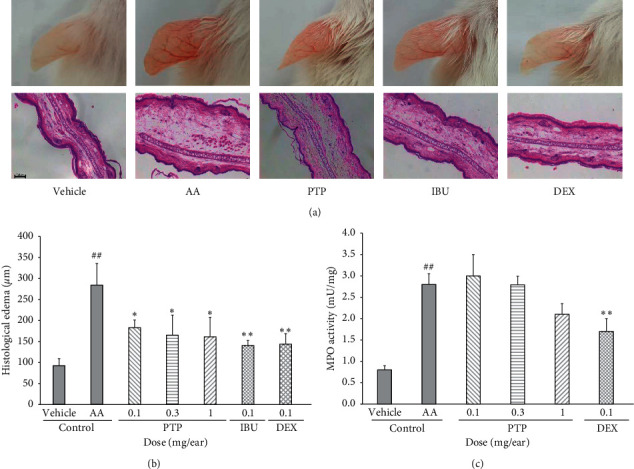
Effect of PTP on arachidonic acid-induced mouse ear edema and MPO activity. Representative images of mouse ears and H & E staining for each experimental group (a). Ear thickness in HPF was measured with the ImageJ software (b). MPO enzymatic activity quantification in response to PTP application (c). The control group was treated with vehicle and AA. All values are reported as the mean ± SD. # indicates a significant difference from the vehicle group (##*P* < 0.01). ^*∗*^ indicates a significant difference from the AA control group (^*∗*^*P* < 0.05, ^*∗∗*^*P* < 0.01). PTP: *Pinus thunbergii* Parl.; DEX: dexamethasone; IBU: ibuprofen, AA: arachidonic acid; MPO: myeloperoxidase; HPF: high-power field.

Our above-described findings were then confirmed *in vivo* by demonstrating that PTP suppresses the degree of edema and ear thickening in an acute inflammatory ear edema murine model. PTP was topically administered at concentrations of 0.1, 0.3, and 1.0 mg/ear, respectively, and ear thickness was measured by inducing inflammation with AA. Compared with the control group, the PTP treatment group exhibited decreased edemas at all tested concentrations. H & E staining was performed to visualize and monitor histological changes, as well as to assess the occurrence and severity of histological edema. The PTP treatment group exhibited decreased swelling at all tested concentrations compared with the control group (Figure 6(a)). PTP inhibited histological edema by 35.6%, 41.9%, and 43.3% at 0.1, 0.3, and 1.0 mg/ear, respectively. Nonetheless, the inhibitory effect of PTP was weaker than that of ibuprofen and dexamethasone at a concentration of 0.1 mg/ear (Figure 6(b)). Furthermore, the degree of tissue damage (i.e., erythema and scab formation, dryness, and gloss) was significantly decreased upon PTP treatment. Myeloperoxidase (MPO) is a proinflammatory enzyme that catalyzes the oxidation of electron donors using hydrogen peroxide as a substrate. Many inflammatory cells, including neutrophils, monocytes, and macrophages, have high levels of MPO [32]. MPO secretion increases when neutrophils are activated due to inflammation and can thus be used as an indicator of tissue inflammation degree. We found that PTP at its highest concentration (1 mg/ear) inhibited MPO enzymatic activity compared to the control group. However, this inhibitory effect was weaker than that of dexamethasone (Figure 6(c)). Collectively, our results indicate that PTP can induce anti-inflammatory effects *in vitro* and *in vivo.*

### 3.7. Analysis of PTP Phytochemicals via LC-MS Analysis

**Figure 7:**
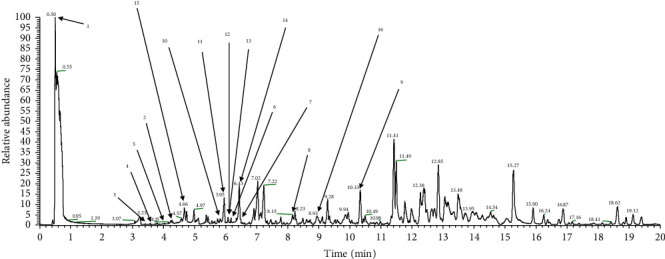
UHPLC-LTQ-Orbitrap chromatogram of PTP extracts. A total of 16 phytochemicals were identified. 1, quinic acid; 2, p-coumaric acid hexoside; 3, gallocatechin; 4, procyanidin B; 5, catechin; 6, quercitrin; 7, isorhamnetin O-glucoside; 8, quercetin (coumaroyl-glucoside); 9, pinocembrin; 10, secoisolariciresinol-diglucoside; 11, lignan derivatives; 12, secoisolariciresinol-glucoside; 13, secoisolariciresinol-xyloside; 14, icariside E4; 15, roseoside; 16, pinellic acid.

The secondary metabolite composition of the analyzed extracts was tentatively characterized based on their retention time, mass spectra, mass fragment pattern, and elemental composition, which were obtained from UHPLC-Q-Orbitrap-MS datasets, as well as from standard compounds, published studies, and web databases such as NIST and MassBank (Figure 7). A total of 16 metabolites were identified in the PTP extracts and categorized into subgroups, including two phenolic acids (quinic acid and p-coumaric acid hexoside), seven flavonoids (gallocatechin, procyanidin B, catechin, quercitrin, isorhamnetin O-glucoside, quercetin coumaroyl-glucoside, and pinocembrin), five lignans (secoisolariciresinol diglucoside, lignan derivatives, secoisolariciresinol glucoside, secoisolariciresinol xylose, and icariside E4), one sesquiterpenoid (roseoside), and one long-chain fatty acid (pinellic acid).

## 4. Conclusions

In summary, our findings confirmed that PTP has a significant anti-inflammatory effect in macrophages. This effect was also confirmed *in vivo*, as PTP suppressed AA-induced ear edema and inhibited MPO enzymatic activity at all treatment concentrations. Moreover, it was found that PTP suppressed the inflammatory response, as demonstrated by intracellular radical, inflammatory enzyme, and cytokine analyses. Moreover, PTP suppressed LPS-induced inflammatory responses by reducing NO, PGE2, and TNF-*α* generation, as well as the expression of proinflammatory mediators, such as iNOS and COX-2, in macrophages. According to our LC-MS analyses, PTP contained large amounts of quinic acid, which has been reported to possess potent antioxidant properties and antineuroinflammatory activity [33]. Furthermore, PTP contains roseoside and icariside E4, which are known to alleviate oxidative stress [34], as well as pinocembrin, which also possesses anti-inflammatory effects [35]. Therefore, our study is the first to identify PTP as a novel potential natural anti-inflammatory agent.

## Data Availability

The data used to support the findings of this study are included in the article.

## Supplementary Materials

Supplementary Materials PTP metabolite identification (ID table, chemical structure of compounds derived from PTP, and reference).
