# Supplementary material for: Pinus thunbergii Parl. Extracts Reduce Acute Inflammation by Targeting Oxidative Stress
Source: Evid Based Complement Alternat Med. 2021 Jan 13;2021:7924645. doi: 10.1155/2021/7924645 (PMC7817271; doi:10.1155/2021/7924645)
Supplement: Supplementary Materials — PTP metabolite identification (ID table, chemical structure of compounds derived from PTP, and reference). [file 7924645.f1.pdf]

ID table

No.	Rt[a] (min)	Tentative Identification	m/z [M+H]$^+$	m/z [M–H]$^-$	M.W.[b]	MS Fragments pattern (m/z)	Molecular Formula	Δ ppm	REF[c]
Phenolic acid									
1	0.51	Quinic acid	215.0527[e]	191.0553	192	191> 173	$C_7H_{12}O_6$	-4.508	STD[d]
2	4.32	*p*-Coumaric acid hexoside	349.1831[e]	325.0930	326	325> 163, 121, 119	$C_{15}H_{18}O_8$	0.490	REF[1]
Flavonoids									
3	3.41	Gallocatechin	307.0811	305.0668	306	305, 289, 219, 181, 167, 137, 125	$C_{15}H_{14}O_7$	0.308	REF[2,3]
4	4.02	Procyanidin B	579.1500	577.1359	578	577> 521, 475, 443, 407, 289, 271	$C_{30}H_{26}O_{12}$	1.353	REF[1,4]
5	4.27	Catechin	291.0863	289.0719	290	289> 245, 203, 191, 163, 137	$C_{15}H_{14}O_6$	1.310	STD[d]
6	6.34	Quercitrin	449.1079	447.0939	448	447> 299, 179, 150	$C_{21}H_{20}O_{11}$	0.585	STD[d]
7	6.49	Isorhamnetin O-glucoside	479.1188	477.1045	478	477> 314, 299, 165	$C_{22}H_{22}O_{12}$	0.731	REF[5,6]
8	8.17	Quercetin (coumaroyl-glucoside)	611.1399	609.1260	610	609> 579, 531, 491, 445, 463, 329, 315, 300, 291	$C_{30}H_{26}O_{14}$	1.001	REF[7]
9	10.31	Pinocembrin	257.0808	255.0663	256	255> 213, 185, 171, 151, 145, 107	$C_{15}H_{12}O_4$	-0.012	STD[d]
Lignan									
10	5.64	Secoisolariciresinol-diglucoside	709.2681[e]	731.2786[f]	686	731> 475, 359, 345, 327, 313, 179, 167	$C_{32}H_{46}O_{16}$	1.767	REF[8-10]
11	5.94	Lignan deriv.	985.4066	491.1930	492	491>473, 327, 313, 149	$C_{25}H_{32}O_{10}$	1.404	REF[8,11]
12	6.07	Secoisolariciresinol-glucoside	547.2153[e]	523.2196	524	523>475, 419, 361, 343, 327, 195, 165	$C_{26}H_{36}O_{11}$	1.558	REF[8,10]
13	6.35	Secoisolariciresinol-xyloside	989.4346[g]	539.2142[f]	494	539> 493, 477, 361, 343, 300, 165	$C_{25}H_{34}O_{10}$	1.540	REF[8.10]
14	6.44	Icariside E4	529.2053[e]	551.2145[f]	506	551> 381, 367, 341, 329, 314	$C_{26}H_{34}O_{10}$	0.744	REF[7,8]
Sesquiterpenoid									
15	4.66	Roseoside	409.1831[e]	431.1925[f]	386	431>385, 367, 223	$C_{19}H_{30}O_8$	0.765	REF[12,13]
Long chain fatty acid									
16	8.91	Pinellic acid	353.2300[e]	329.2337	330	329> 229, 211, 179, 171	$C_{18}H_{34}O_5$	0.293	REF[14,15]

[a] Retention time; [b] Molecular weight; [c] Reference; [d] Standard; [e] [M+Na]; [f] [M+FA-H]; [g] [2M+H]

Compound chemical structure derived from *PTP*

No.1 Quinic acid

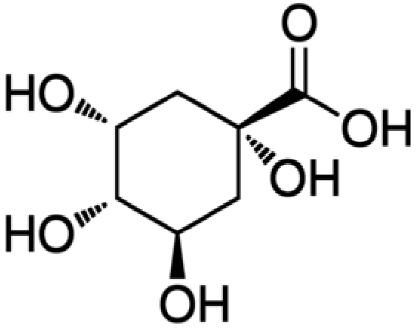

No.2 *p*-Coumaric acid hexoside

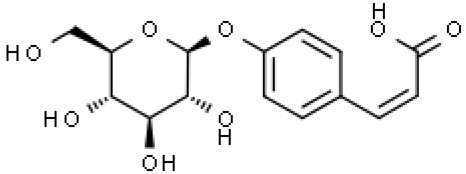

No.3 Gallocatechin

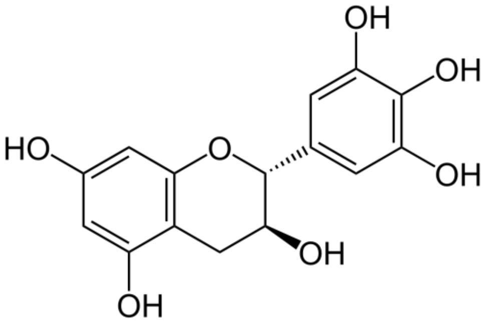

No.4 Procyanidin B

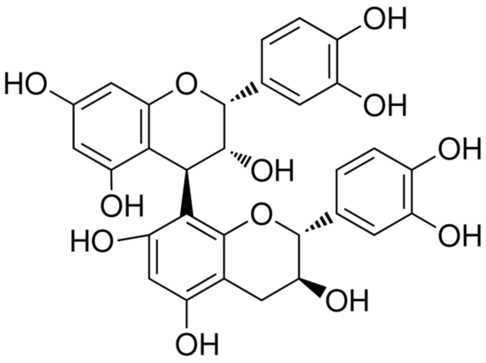

No.5 Catechin

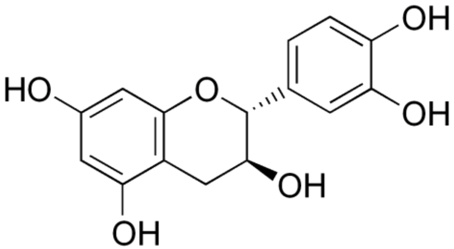

No.6 Quercitrin

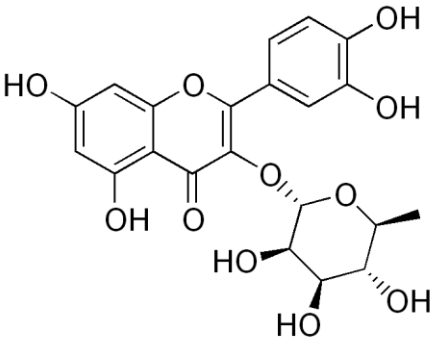

No.7 Isorhamnetin O-glucoside

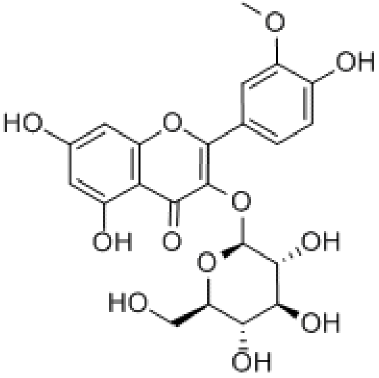

No.8 Quercetin (coumaroyl-glucoside)

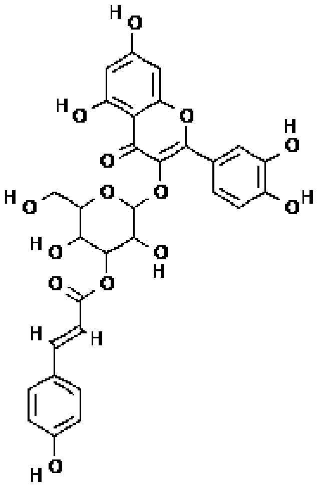

Compound chemical structure derived from *PTP*

No.9 Pinocembrin

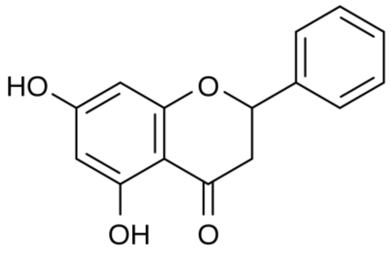

No.10 Secoisolariciresinol-diglucoside

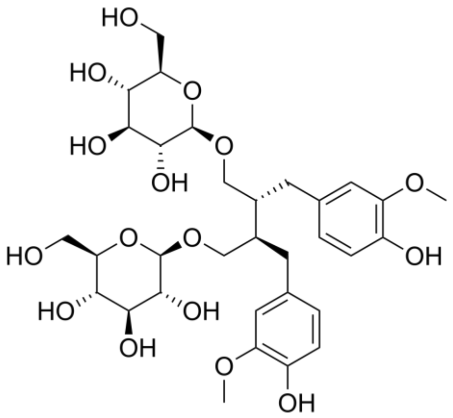

No.11 Lignan deriv.

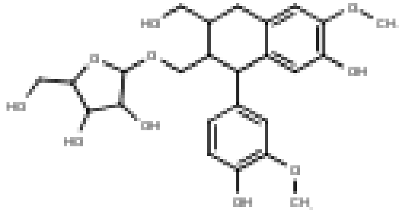

No.12 Secoisolariciresinol-glucoside

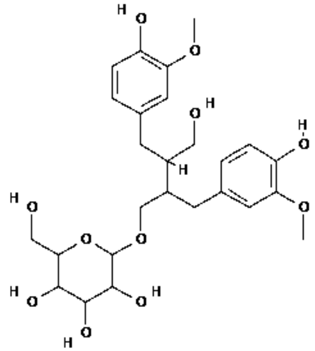

No.13 Secoisolariciresinol-xyloside

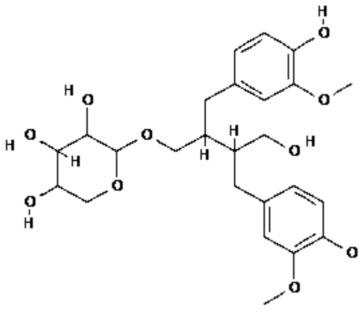

No.14 Icariside E4

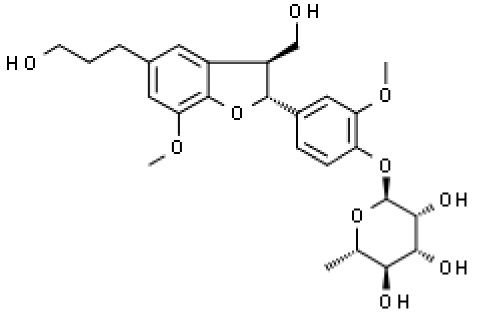

No.15 Roseoside

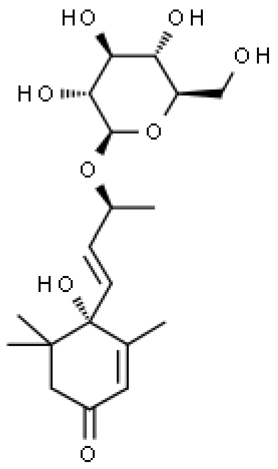

No.16 Pinellic acid

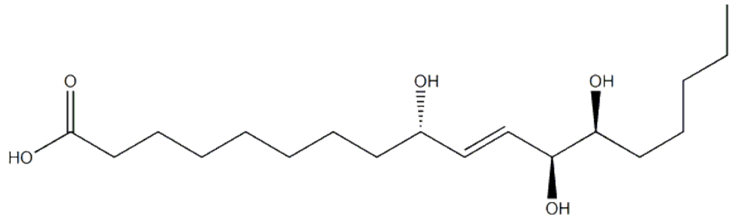

Reference

REF[1] Mekky, Reham Hassan, et al. "Phenolic compounds from sesame cake and antioxidant activity: a new insight for agri-food residues' significance for sustainable development." *Foods* 8.10 (2019): 432.

REF[2] Yuzuak, Seyit, James Ballington, and De-Yu Xie. "HPLC-qTOF-MS/MS-Based Profiling of Flavan-3-ols and Dimeric Proanthocyanidins in Berries of Two Muscadine Grape Hybrids FLH 13-11 and FLH 17-66." *Metabolites* 8.4 (2018): 57

REF[3] Lundgren, Lennart N., and Olof Theander. "Cis-and trans-dihydroquercetin glucosides from needles of *Pinus sylvestris*." *Phytochemistry* 27.3 (1988): 829-832.

REF[4] Li, Ying-Ya, et al. "Pine bark extracts: nutraceutical, pharmacological, and toxicological evaluation." *Journal of Pharmacology and Experimental Therapeutics* 353.1 (2015): 9-16.

REF[5] Slimestad, R. "Flavonoids in buds and young needles of *Picea*, *Pinus* and *Abies*." *Biochemical systematics and ecology* 31.11 (2003): 1247-1255.

REF[6] Lei, Zhentian, et al. "Metabolomics of Two Pecan Varieties Provides Insights into Scab Resistance." *Metabolites* 8.4 (2018): 56.

REF[7] Chen, Guan-Heng, et al. "Characterization of vasorelaxant principles from the needles of *Pinus morrisonicola Hayata*." *Molecules* 23.1 (2018): 86..

REF[8] Li, Bo, et al. "Chemical constituents and biological activities of *pinus* species." *Chemistry & biodiversity* 10.12 (2013): 2133..

REF[9] Yashunsky, D. V., et al. "Analysis of content of (–)-secoisolariciresinol and related polyphenols in different morphological parts and anatomical structures of larch wood from *Siberia*." *Russian Chemical Bulletin* 63.11 (2014): 2571-2576.

REF[10] Quartieri, Andrea, et al. "Detection of novel metabolites of flaxseed lignans in vitro and in vivo." *Molecular nutrition & food research* 60.7 (2016): 1590-1601.

REF[11] Fischer, Ulrike Anna, et al. "Determination of lignans in edible and nonedible parts of pomegranate (*Punica granatum L.*) and products derived therefrom, particularly focusing on the quantitation of isolariciresinol using HPLC-DAD-ESI/MS n." *Journal of agricultural and food chemistry* 60.1 (2012): 283-292.

REF[12] Sun, Liqiong, Shutian Tao, and Shaoling Zhang. "Characterization and quantification of polyphenols and triterpenoids in thinned young fruits of ten pear varieties by UPLC-Q TRAP-MS/MS." *Molecules* 24.1 (2019): 159.

REF[13] Shen, YanBo, and Minoru Terazawa. "Dihydroroseoside, a new cyclohexanone glucoside, from the leaves of shirakamba (*Betula platyphylla Sukatchev var. japonica Hara*)." *Journal of wood science* 47.2 (2001): 145-148.

REF[14] Ruan, Jingya, et al. "Comprehensive Chemical Profiling in the Ethanol Extract of *Pluchea indica* Aerial Parts by Liquid Chromatography/Mass Spectrometry Analysis of Its Silica Gel Column Chromatography Fractions." *Molecules* 24.15 (2019): 2784.

REF[15] Peng, Wan-Xi, et al. "Determination of biomedicine resource of benzene/ethanol extractives of masson pine (*Pinus massoniana L.*) wood by Py-GC/MS." *2008 2nd International Conference on Bioinformatics and Biomedical Engineering*. IEEE, 2008.
